# Preparing controlled low strength materials with cement-treated construction waste clay improved by sodium hexametaphosphate

**DOI:** 10.1371/journal.pone.0314077

**Published:** 2025-02-06

**Authors:** Chenhao Li, Tinglong Xie, Jianwen Ding, Jian Tang, Mengying Gao

**Affiliations:** 1 Institute of Geotechnical Engineering, School of Transportation, Southeast University, Nanjing, China; 2 Jiangsu Provincial Construction Group Co., Ltd., Nanjing, China; Kobe University: Kobe Daigaku, JAPAN

## Abstract

To solve the disposal of large quantities of construction waste clay, this study proposes a new method for preparing controlled low strength materials (CLSM). Flow tests, unconfined compressive strength (UCS) tests, hydraulic conductivity tests and scanning electron microscope (SEM) analyses were performed on cement-treated construction waste clay with different additive content (e.g. sodium hexametaphosphate (SHMP), water glass, and phosphogypsum (PG)). The influence of additive content on the mechanical and microstructural properties of cement-treated clay-based CLSM was analyzed. The results indicated that the SHMP greatly enhanced the flowability of samples, adding 1%SHMP increased the fluidity of the sample by more than 80%, whereas 5% water glass had negligible effect. Additionally, the 10% PG improved the flowability retention, making it have higher flowability after 30 mins (more than 200 mm). SHMP interacted with Ca^2+^, significantly influencing the cement hydration; notably, 1% content resulted a notable reduction of samples from 167.5 kPa to 21.5 kPa at 1 day. Although increasing SHMP content improved the early strength, it led to a decrease in later strength, with the maximum late strength observed at 2% SHMP. Both PG and water glass also contributed to late strength enhancement, though higher SHMP levels diminished their effects. While SHMP markedly improved permeability resistance (less than 8 × 10^−8^ cm/s after 28d), hydraulic conductivity showed minimal variation with increased dosage. The combination of SHMP, PG and water glass effectively enhances the flowability and strength of clay-based CLSM at low water content, solving the contradiction between fluidity and strength. This promotes the sustainable development of green building materials.

## 1. Introduction

The CLSM proposed by the American Concrete Institute (ACI), is a backfill material with self-leveling and self-compacting properties [1]. Its high flowability makes it a suitable material for various applications, including trench backfilling and void filling [2, 3]. CLSM typically consist of fly ash, fine aggregate, water, cement, and chemical admixture [4, 5]. Notably, CLSM does not have explicit requirements for raw materials, and any non-standard materials that meet the expected performance can be used to produce CLSM [6].

Currently, most construction waste soil is disposed of by external transportation. It increases project costs and wastes land resources [7, 8]. To promote resource utilization of construction waste soil, researchers have explored the possibility of utilizing it to produce CLSM. However, the high hydrophilicity and low fluidity of clay limit its use as aggregates. To overcome this challenge, relevant studies have been conducted, such as using excavated soil as a partial replacement for sand to produce CLSM for backfilling [9, 10]. A properly designed sand-to-soil ratio can meet engineering requirements, but higher soil content can lead to reduced strength and flowability. Although the clay enhanced the water retention, more water was required to meet the flowability requirements [11]. Additionally, the higher water content increased the risk of segregation and bleeding, leading to a decrease in long-term strength [8, 12, 13]. Therefore, to better utilize construction waste soil in engineering applications, the contradiction between strength and fluidity of clay in CLSM preparation is an urgent problem to be solved.

The sustainable utilization of waste has been identified as one of the major research topics in the field of sustainable development within the construction industry [14–18]. Currently, many new types of green building materials have been developed and applied, utilizing various materials such as fibers [19, 20], nanomaterials [21], and biomaterials [22, 23]. Ghasemzadeh et al. [24, 25] explored the application of Persian gum in the stabilization of low-plasticity clay. Through UCS tests, direct shear tests, and SEM, they verified the excellent performance of Persian gum in binding soil particles, filling voids, enhancing thermal stability, mitigating soil fluctuations, and forming larger aggregates. Kakroudi et al. [26] investigated the effects of Nano silica and basalt fiber content, curing time, and freeze-thaw cycles on the static and dynamic properties of soil samples. Through a series of laboratory tests, they found that a combination of 1% fiber and 10% Nano silica achieved optimal soil reinforcement, resulting in maximum cohesion and friction angle of 90 kPa and 37.8°, respectively. Hence, it is effective to consider adding different additives to improve the performance of the original material.

SHMP is a commonly used soil particle dispersant in geotechnical tests, especially for clay particles. It could mitigate the flocculation phenomenon of the soil and transform soil particles from disordered to ordered arrangement, thereby improving its strength, compressibility, permeability, and other engineering properties [27–29]. The surface of soil particles was adsorbed by phosphate ions, forming a protective film that prevents particle contact (i.e. steric hindrance effect). Additionally, phosphate ions can increase the negative surface potential of soil particles, enlarge the double electric layer thickness, and enhance interparticle repulsion [30–32]. These two effects make soil particles in a dispersed state. Adding SHMP is an effective method to improve the flowability of CLSM. However, the dispersion and mechanical effects of SHMP in cement-based materials are need to further identified.

PG is a by-product generated during the production of phosphoric acid and its main component is CaSO_4_·2H_2_O [33]. It can react with cement to generate ettringite (AFt), which fills the large pore structure within the soil mass, leading to a reduction in porosity. This has a positive effect on promoting strength development and decreasing permeability [34–36]. In addition, as the formed AFt covers the surface of cement particles, it retards cement hydration, thereby exhibiting a retarding effect [37, 38]. Sodium silicate is a water-soluble silicate, and its aqueous solution is known as water glass. The hydrolysis of water glass generates silicic acid that reacts with the Ca(OH)_2_ produced during cement hydration. It disrupts the hydrolysis equilibrium of tricalcium silicate (C_3_S) and dicalcium silicate (C_2_S), leading to increased hydration and a faster cement setting rate. This results in improved early strength development [39].

Due to the high clay content in construction waste soil, using it entirely as aggregate necessitates the addition of a significant amount of water to meet performance requirements, which adversely affects the development of long-term strength. Moreover, there are currently no precedents for using SHMP, water glass, and PG to prepare controllable low-strength materials, though these additives have demonstrated efficacy in regulating material working performance.

This study aims to explore the potential application value of construction waste soil at to prepare CLSM. The results also reveal that there is an optimum proportion for combined SHMP with PG and water glass, which provides a reference for the determination of proportions in relevant applications. Firstly, the three materials (i.e. SHMP, water glass and PG) were combined according to predefined ratios to obtain the samples. Next, a series of flow tests, UCS tests were performed on the CLSM samples to investigate the effects of different additives on flowability, setting time, strength, and age development. Finally, the underlying mechanisms of samples were revealed through SEM tests. Moreover, the application of the research findings in practical engineering can promote the extensive utilization of construction waste as fill material, thereby reducing the storage of waste soil and Industrial By-Products (IBPs) and yielding significant economic and environmental benefits.

## 2. Materials and methods

### 2.1 Materials

**Fig 1:**
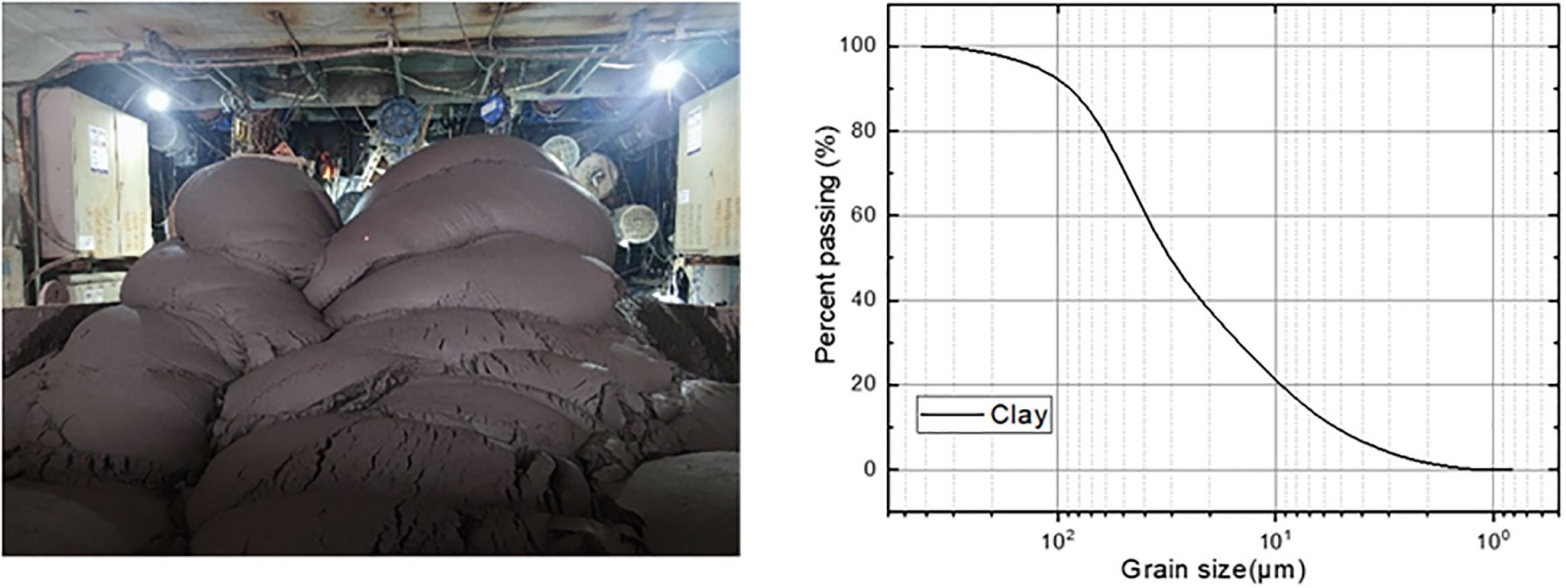
(a)Raw soil; (b) The grain size distribution.

The soil used in this study was sampled from Nanjing, Jiangsu, China, which was generated during the pipe-jacking construction. The grain size distribution of soil is shown in Fig 1. The liquid limit (*w*_L_) and the plastic limit (*w*_P_) of the soil were determined using a fall cone penetrometer according to the standard procedure specified in BS1377-2:1990. Table 1 presents the basic physical properties of the soil. The values of *w*_L_ and *w*_P_ were 33.6% and 15.7%, respectively. According to the Unified Soil Classification System [40], the soil can be classified as CL clay.

**Table 1:** Basic physical properties of soil.

Initial Moisture/%	Density/ (g/ cm^3^)	Plastic limit/%	Liquid limit/%	Plasticity index
24.8	1.79	17.9	33.6	15.7

**Fig 2:**
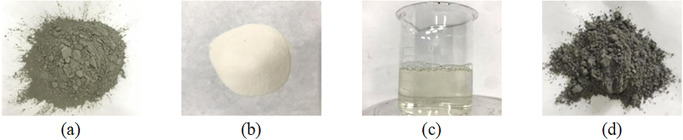
(a) Cement; (b) SHMP; (c) Water glass; (d) PG.

The cement used is M•32.5 from Conch Cement Co., LTD. Masonry cement exhibits favorable workability and water retention characteristics, albeit with relatively low strength, making it suitable for the requirements of CLSM. The SHMP is sourced from Tianjin Dinasheng Xin Co., LTD. It has an analytical grade purity. The water glass is supplied by Jiashan County Yourui Refractory Co., LTD., featuring a Baume degree 38.5 and a modulus of 3.30. The materials used in the test are shown in Fig 2 and the composition is shown in Table 2.

**Table 2:** Chemical composition of test materials (mass fraction)/%.

Material	CaO	SiO_2_	Fe_2_O_3_	SO_3_	Al_2_O_3_	MgO	Loss
Raw soil	4.1	64.65	6.69	0.61	17.00	2.14	4.81
Cement	65.75	18.26	4.42	4.86	3.20	/	3.51
PG	34.71	8.41	0.37	51.91	1.28	0.67	2.65

### 2.2 Mixing design and testing methods

To investigate the effect of these materials on the engineering performance of CLSM, and determine the optimal combination of additives, experiments were conducted using different dosage ratios. First, a predetermined amount of granular SHMP was dissolved in water using a magnetic stirrer to prepare an SHMP solution. Based on preliminary experiments, the cement content was fixed at 15% (as a percentage of the dry soil mass) with *w*_0_ = 1.4*w*_L_. Note that *w*_0_ was the initial water content. The *w*_L_ was the water content at liquid limit. Subsequently, pour the prepared SHMP solution into the measured soil and materials, and thoroughly mix using a blender.

The detailed design is shown in Table 3. The mixed ratio can be expressed as L1P10G5. Note that, L1 indicates the mass of SHMP (10 g), accounting for 1% of the mass of dry soil (1000 g); P10 denotes the mass of PG (15 g), accounting for 10% of the mass of cement (150 g); G5 represents the mass of water glass (7.5 g), accounting for 5% of the mass of cement.

**Table 3:** Program of this study.

Cement (%)	Water content	SHMP (%)	PG (%)	Water glass (%)
15%	1.4 *w*_L_	0	0 10 20	0
		1		5
		2		10
		3		15

#### 2.2.1 Flow test

**Fig 3:**
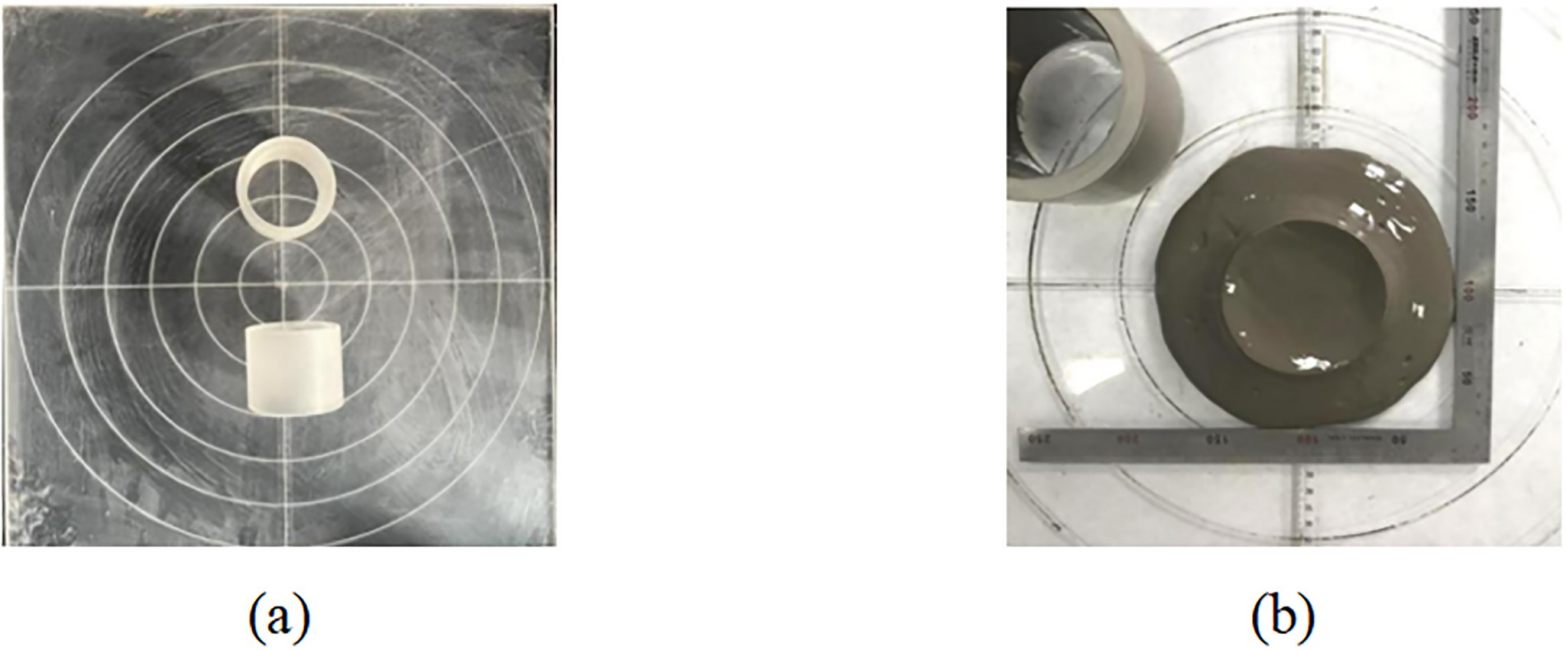
(a) Measuring device; (b) Measuring method.

Flowability is a key indicator of CLSM, and good flowability ensures self-compactness during backfilling. The flowability was determined following T/CES 1037–2022 [41]. Firstly, a plexiglass cylinder with a height and inner diameter of 80 mm was placed at the center of a plexiglass plate. Next, the prepared mixed CLSM was poured into this cylinder. Then, it was rapidly lifted vertically, and after waiting for approximately 30s, the average diameter of the spread mixture in two perpendicular directions was measured using a steel ruler, as shown in Fig 3. To ensure the reliability of the experiment, 2 to 3 parallel tests were conducted for each soil sample group, and the average value of each experiment was taken as the flow value of the modified soil sample.

#### 2.2.2 UCS and hydraulic conductivity tests

**Fig 4:**
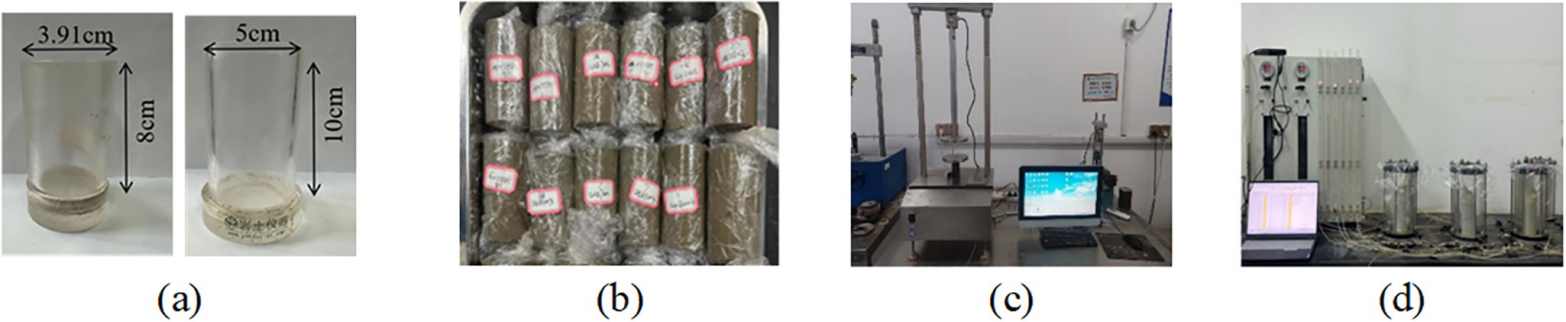
(a) Molds; (b) Samples; (c)(d) Test devices.

Cylindrical molds were used to prepared the samples, as shown in Fig 4(a). Firstly, the fresh CLSM was poured into these cylindrical molds. After obtaining a certain strength, the samples were demolded and sealed with plastic wrap to prevent water loss. Then the samples were put into the curing room for curing. Note that all the samples were cured at a temperature of 20 ± 2°C and a relative humidity of 95%. When the predetermined curing time (i.e. 1d, 3d, 7d, 28d and 60d) was reached, the samples (3.91cm×8cm) were tested for UCS according to ASTM D2166. The UCS was carried out using an automatic loading machine with a loading rate of 1 mm/min (Fig 4(c)). The specimens with 50 mm in diameter and 100 mm in height were cured for 7 and 28 days, respectively. The hydraulic conductivity was measured by a flexible-wall permeameter according to ASTM D5084-16a 2016 (Fig 4(d)).

#### 2.2.3 SEM analysis

To examine the microstructure and hydration products formed, SEM was performed on the 7-day and 28-day cured samples. After the UCS tests, powdered samples and cube samples (1 cm × 1 cm × 1 cm) were obtained from the failure surface of the samples at the corresponding age, and then freeze-dried in the freeze-drying machine. The SEM analysis was conducted using a TESCAN SEM. Before testing, a platinum layer with a thickness of 20–30 nm was applied to all samples to facilitate SEM imaging.

## 3. Results and discussion

### 3.1 Flow test

As shown in Table 4, the flowability of L0P0G0 was 91 mm, indicating poor flowability of the mixture at this ratio. With the increase in SHMP, the flowability of each group exhibited a significant increase. When the SHMP contents were 1%, 2%, and 3%, the flowability was 167 mm, 250 mm, and 253 mm, respectively. This can be attributed to the excellent deflocculating effect of SHMP on soil particles [32]. When the SHMP dissolved in water, the numerous negatively charged polyphosphate anions were released. These anions are adsorbed onto soil particle surfaces, generating electrostatic repulsion. Furthermore, owing to its large cyclic phosphate structure, SHMP can cause steric hindrance. The combined effects of electrostatic repulsion and steric hindrance gave SHMP great dispersing properties [31, 32]. As the SHMP content continued to increase (1% to 3%), the flowability kept rising. However, once the SHMP content reached 2%, the improvement in flowability became less significant. This may because most of the water enclosed in the soil particles had already been released. Moreover, the viscosity of the SHMP solution may also cause a reduction in the flowability of CLSM.

**Table 4:** Flowability of mixture with different SHMP content.

Mixture	SHMP (%)	Flowability (mm)
L0P0G0	0	91
L1P0G0	1	167
L2P0G0	2	250
L3P0G0	3	253

**Fig 5:**
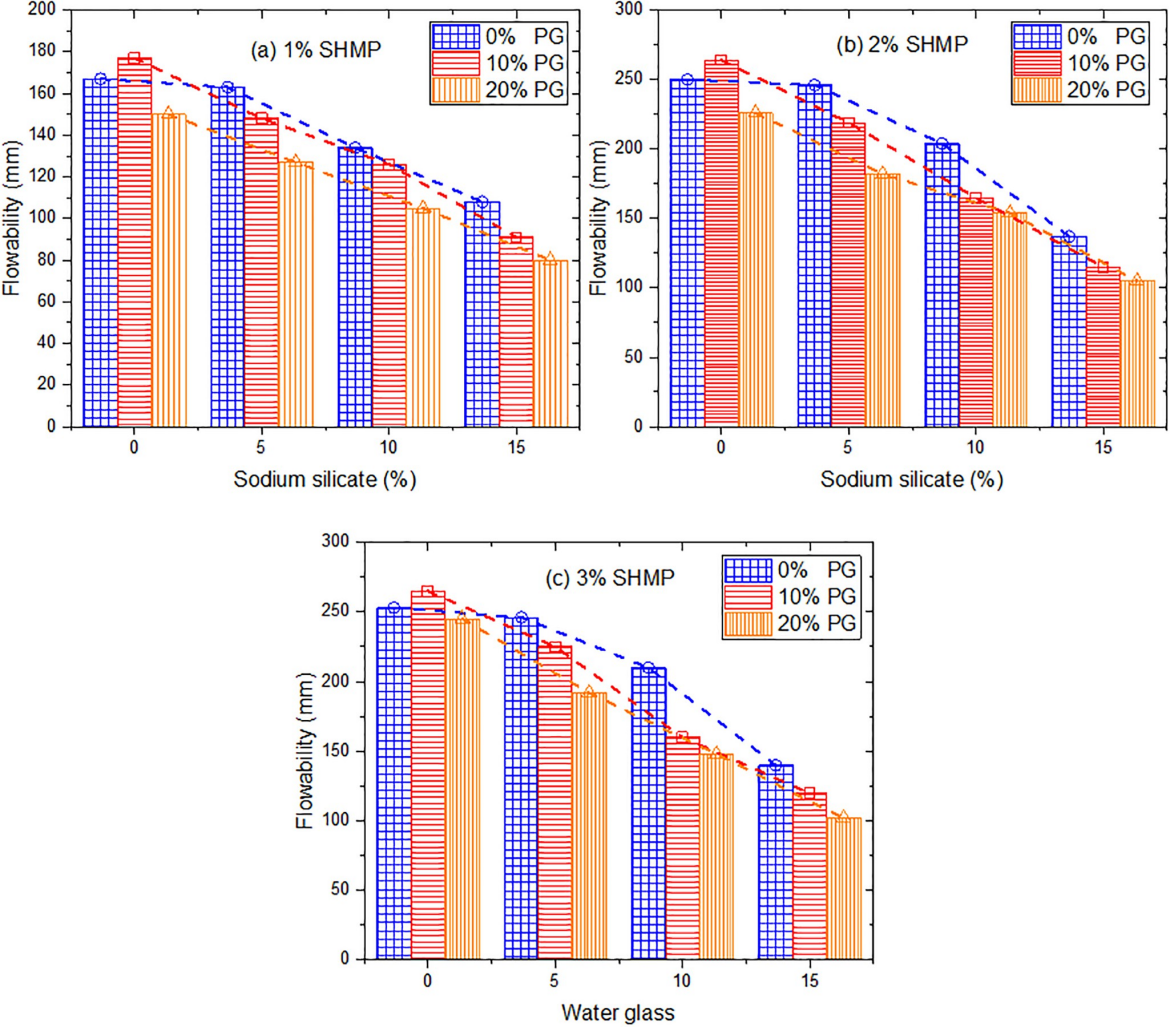
Variation of flowability with different additives.

Fig 5 illustrates the influence of the water glass and PG content on the flowability of the mixture. The flowability of the mixture steadily decreased with the increasing in water glass. When the water glass content was 15%, the flowability of the samples decreased by more than 50%. The hydration balance of cement was disrupted by the addition of water glass, which resulted in the formation of a considerable amount of C-S-H and decreased flowability [39]. In the sample without added PG, the 5% water glass had little effect on the flow properties of the mixture. This may be because the addition of a small amount of water glass could effectively inhibit the hydration of tricalcium aluminate (C_3_A) [42, 43]. On the other hand, water glass contained a certain amount of water, and it could improve flowability. However, after adding PG, Ca^2+^ was introduced into the system and reacted quickly with sodium silicate, resulting in a decrease in flowability. Noted that when no water glass was added, the addition of PG led to a minor improvement in flowability for each group. This may be due to the retarding effect of PG on the hydration of cement [37]. Meanwhile, the presence of fine PG particles enhanced the powder packing, releasing entrapped water. More free water was available for lubrication to enhance flowability [44]. However, the addition of PG effectively reduced the water content, and the reaction between PG and C_3_A consumed water; thus, when the PG addition reached 20%, flowability began to decrease.

**Fig 6:**
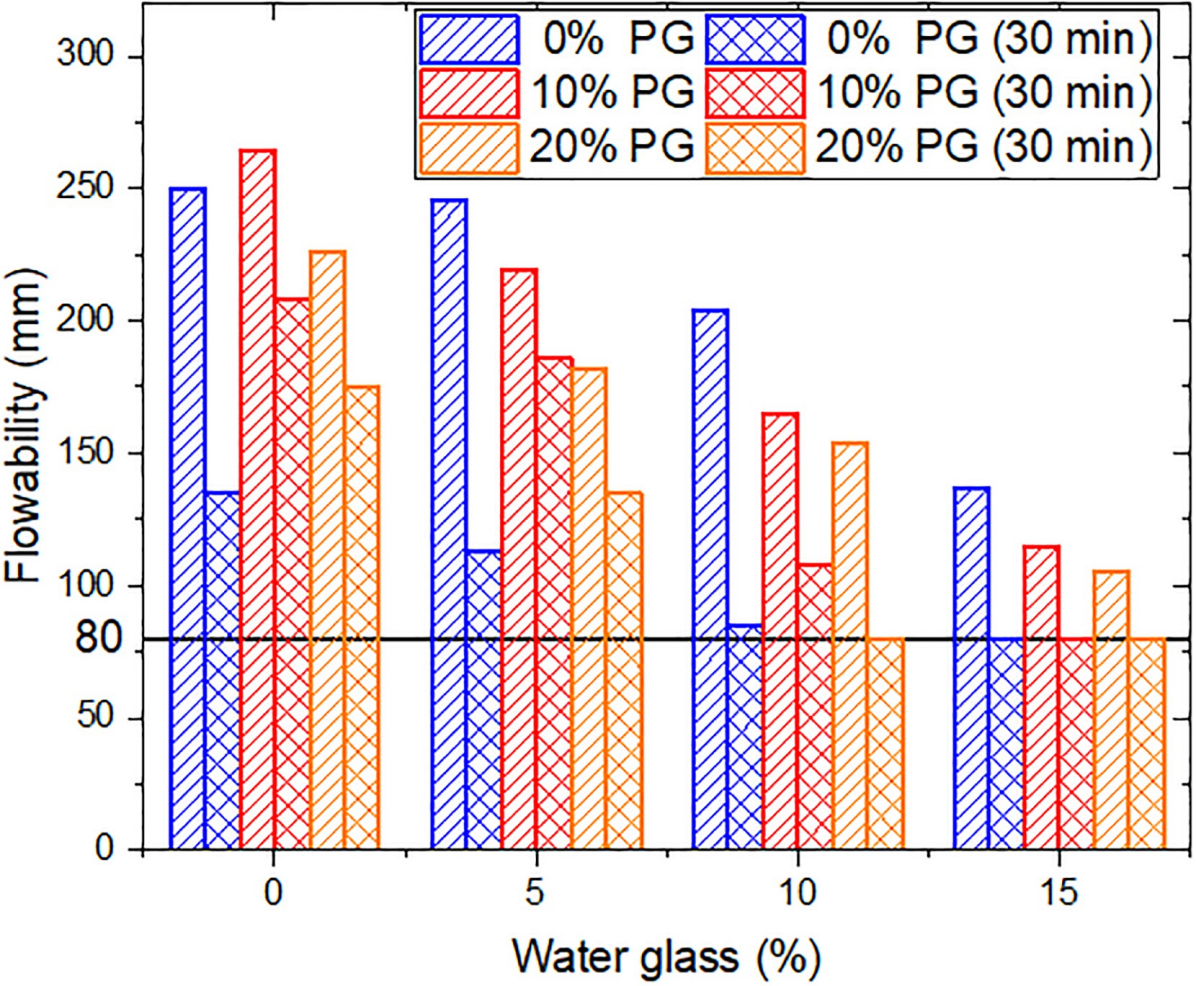
Flowability variation after 30mins.

The setting time is crucial for planning construction operations for structural fills. Fig 6 depicts the flowability variation after 30 mins in the group containing 2% SHMP. It can be observed that there was a decrease in flowability for all groups with time. In the group without PG, the decline was approximately 50% or more. For example, the flowability of L2P0G0 decreased from 250 mm to 135 mm after 30 mins, while L2P0G5 decreased from 246 mm to 113 mm. Similarly, groups with 10% and 15% water glass added experienced reductions to 85 mm and 80 mm, respectively, and the samples were almost non-flowable. The addition of PG could inhibit cement hydration, significantly reducing the loss in flowability [38]. Specifically, with the addition of 10%PG, both L2P10G0 and L2P10G5 showed a decrease in flowability of less than 20% after 30 mins. However, the 10% or more water glass resulted in a near loss of flowability for these samples within the same time frame. This phenomenon can be attributed to cement hydration and the reaction of sodium silicate with Ca^2+^ in the system, leading to water consumption and the generation of gelling substances. PG can release calcium ions, which react with water glass, consuming a significant amount of water and resulting in a substantial reduction in the material’s flowability.

### 3.2 UCS

**Fig 7:**
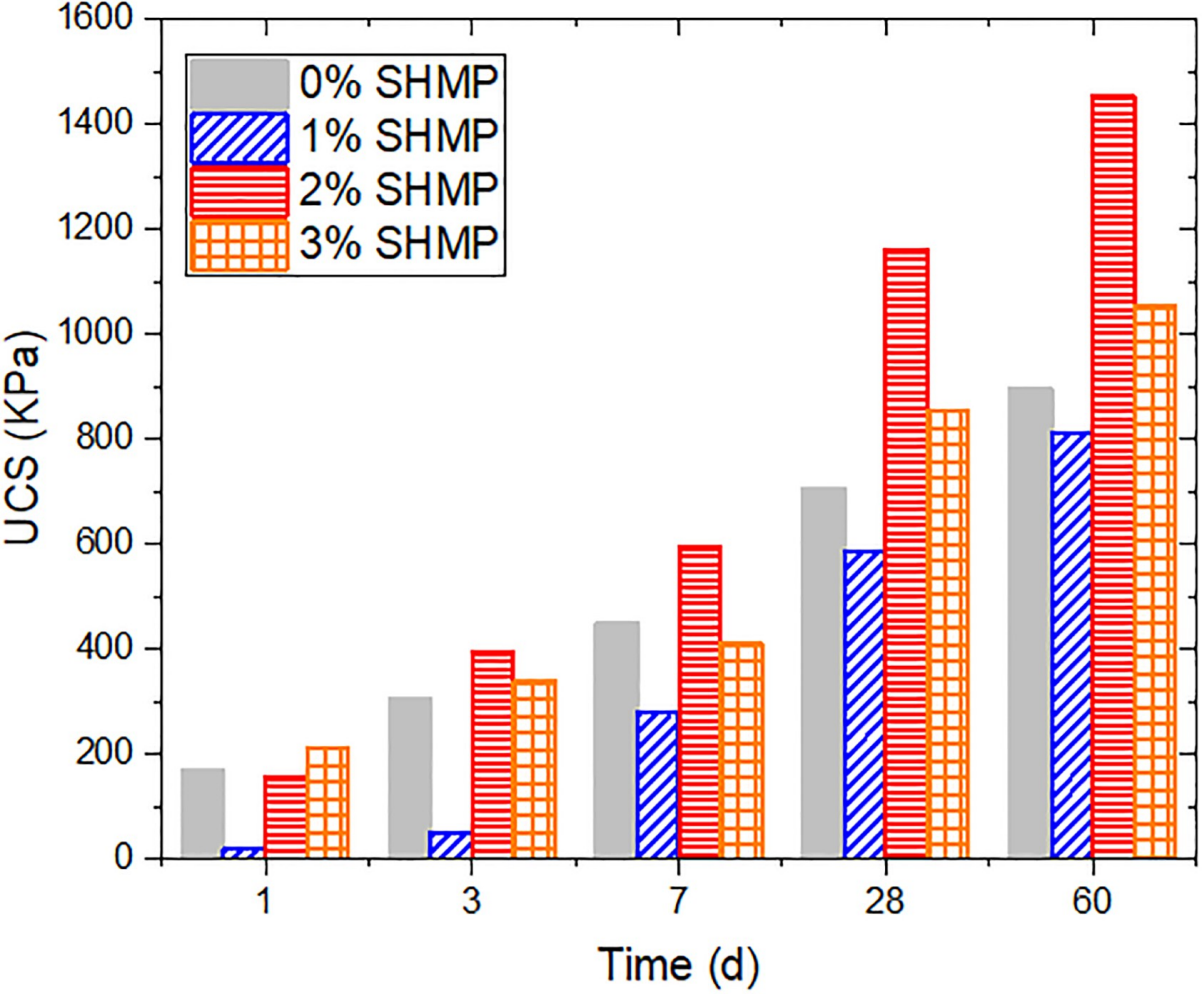
Variation of UCS with SHMP content.

UCS is another indicator for assessing the engineering performance of CLSM. As shown in Fig 7, there was a significant increase in UCS with the curing time, due to the increased hydration products. The strength of the treated soil with the 1% SHMP significantly decreased during the early curing period, especially 7 days before. The UCS at 1 day and 3 days were 26.2 kPa and 40.3 kPa, respectively. The strength was significantly lower than those of the sample without SHMP. This can be attributed to the absorption of SHMP on the surface of cement particles, and formed a protective layer by chelating Ca^2+^. This protective layer retarded the hydration process of cement [45]. The inhibitory effect of SHMP on cement hydration was reported by Zhang et al [46].

**Fig 8:**
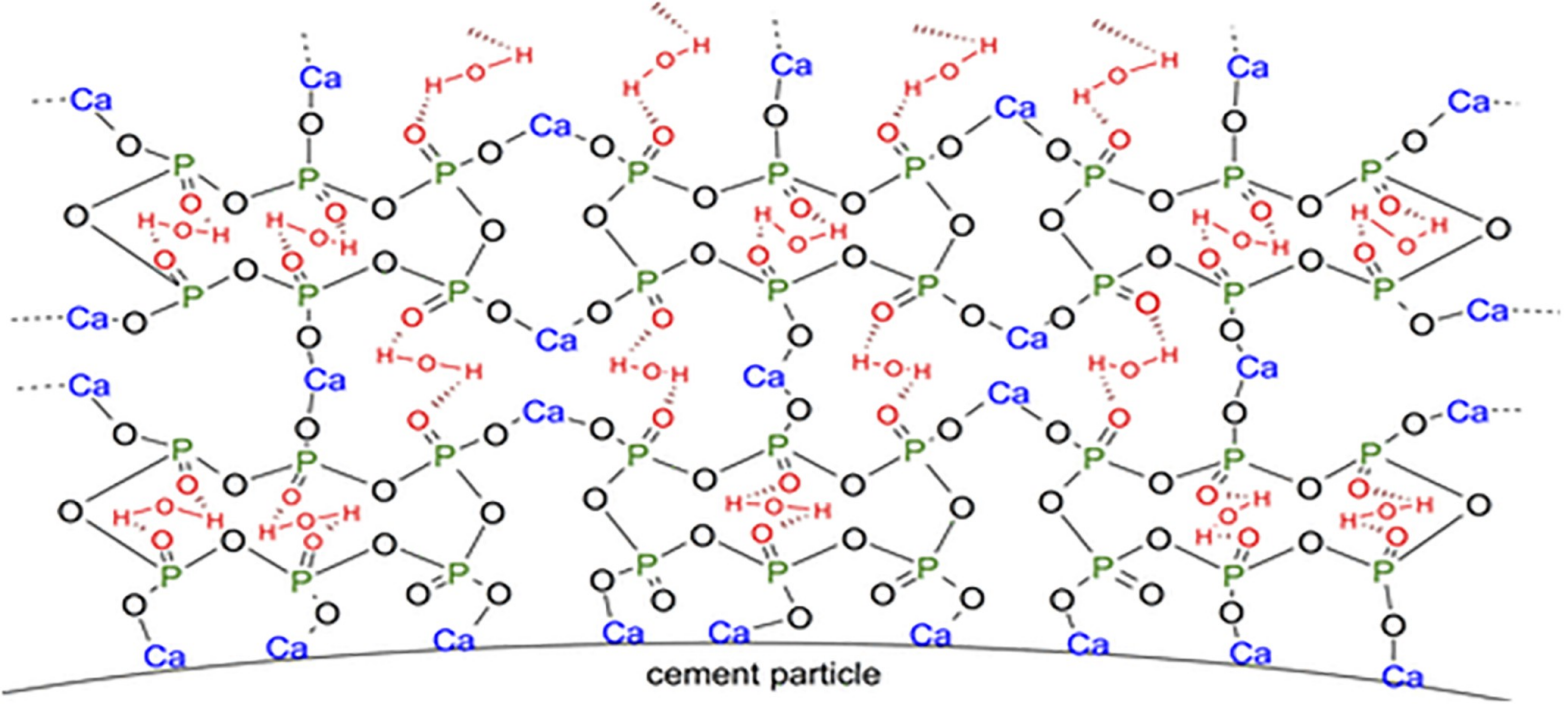
Effect of the adsorbing behavior of phosphate retarders on hydration of cement Paste [29].

However, as the curing time increased, more cement participated in the hydration reaction, resulting in a substantial increase in UCS after 7 days. The strength of the sample reached over 60% of that without SHMP. After 60 days, although the strength of L1P0G0 still did not exceed that of L0P0G0, the UCS of these two were very close. The 2% or more SHMP could effectively improve the early strength of the samples. When the SHMP content increased from 1% to 2%, the 1-day UCS significantly rose from 21.5 kPa to 155.4 kPa. When the SHMP content reached 3%, the 1-day UCS was 213.2 kPa, surpassing that of the L0P0G0 group. With the gradual increase of SHMP content, more SHMP participated in the chelation reaction with Ca^2+^, forming a calcium-based phosphate salt on the surface of cement particles [43]. The unique structure of SHMP, wrapped more water molecules into the net structure (Fig 8), which reduced the amount of free water in the samples and increased their strength. A thicker complexation layer implied that cement was less likely to undergo hydration reactions, resulting in lower strength for the L3P0G0 group than the L2P0G0 group after 3 days. On the other hand, the combined action of the gelling substance formed by the complexation of SHMP with Ca^2+^ and the hydration products of cement significantly increased the UCS of the later-stage samples.

**Fig 9:**
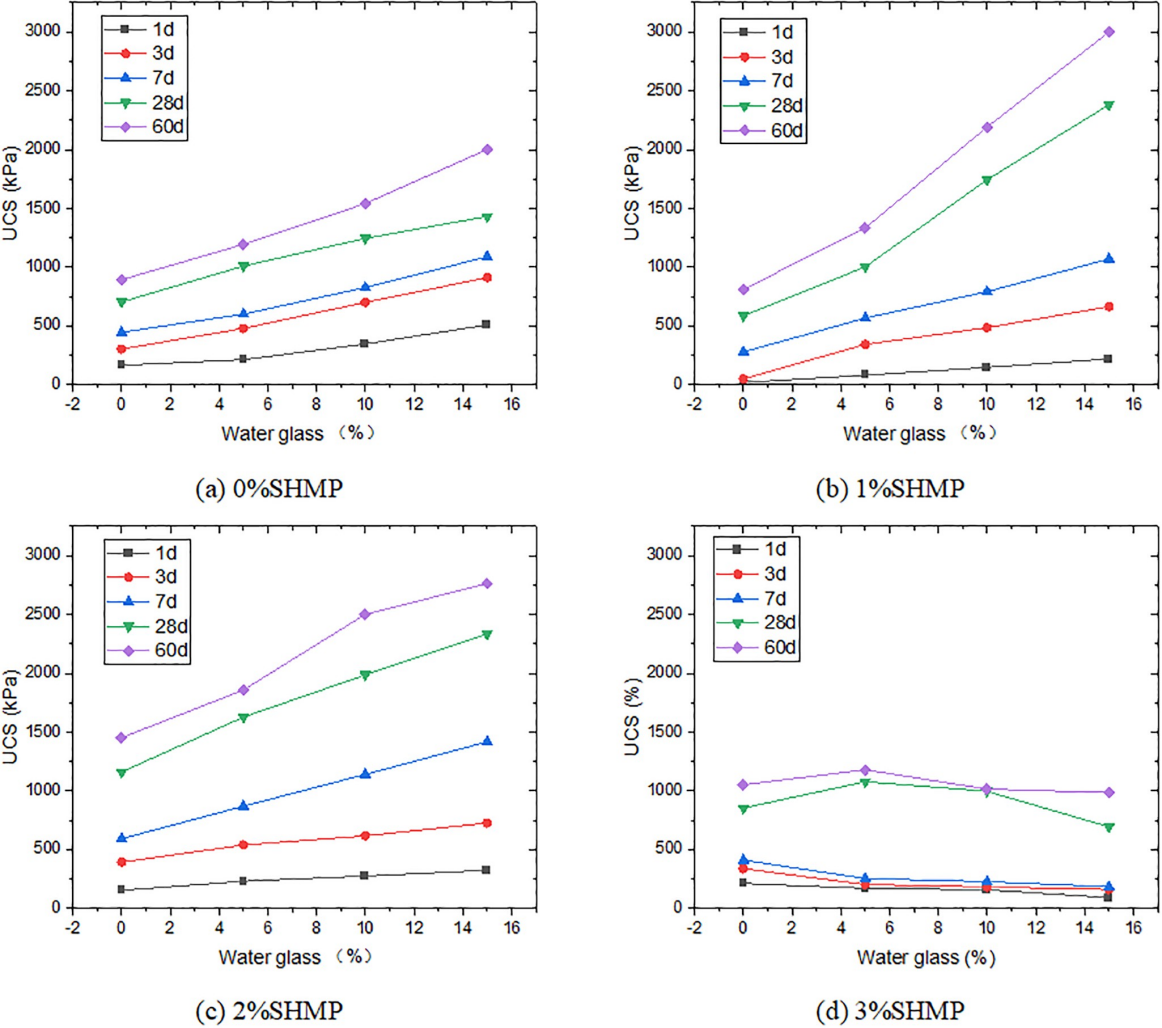
Variation of UCS with water glass and SHMP content.

When the content of SHMP was below 3%, the UCS at all ages increased with water glass, particularly in the early stages, as depicted in Fig 9. The L1P0 group experienced a remarkable increase in strength, with 1-day and 3-day values rising from 21.5 kPa to 221.1 kPa and from 50.8 kPa to 621.1 kPa, respectively, as water glass content increased from 0% to 15%. The early strength of the other two groups (L0P0 and L2P0) also increased by more than double. This is attributed to the promotion of C_3_S hydration by water glass, resulting in the generation of a large amount of C-S-H gel, which improved the strength of the samples [39, 47]. As the curing time progressed, the contribution of water glass to strength weakened due to continuous cement hydration. However, the results showed that the addition of water glass resulted in a stable increase in strength that did not significantly decrease over time. The positive impact on strength still can been seen even after 60-day curing period. In L1P0 and L2P0 groups, the increase in strength was more pronounced in the later stages, as calcium-based phosphate salt formed by SHMP and Ca^2+^ made a denser structure with the cement hydration products. Nevertheless, when the SHMP content exceeded 3%, the addition of water glass had an adverse effect. The adsorption of SHMP onto cement particle surfaces resulted in a decrease in the contact area between water and cement, leading to the suppression of water glass’s ability to promote cement hydration. Moreover, water glass could also inhibit the hydration of C_3_A [42], while introducing free water, resulting in a decrease in the UCS before 7 days. In the later stages, because most Ca^2+^ had been complexed, the contribution of water glass to strength enhancement became very limited.

**Fig 10:**
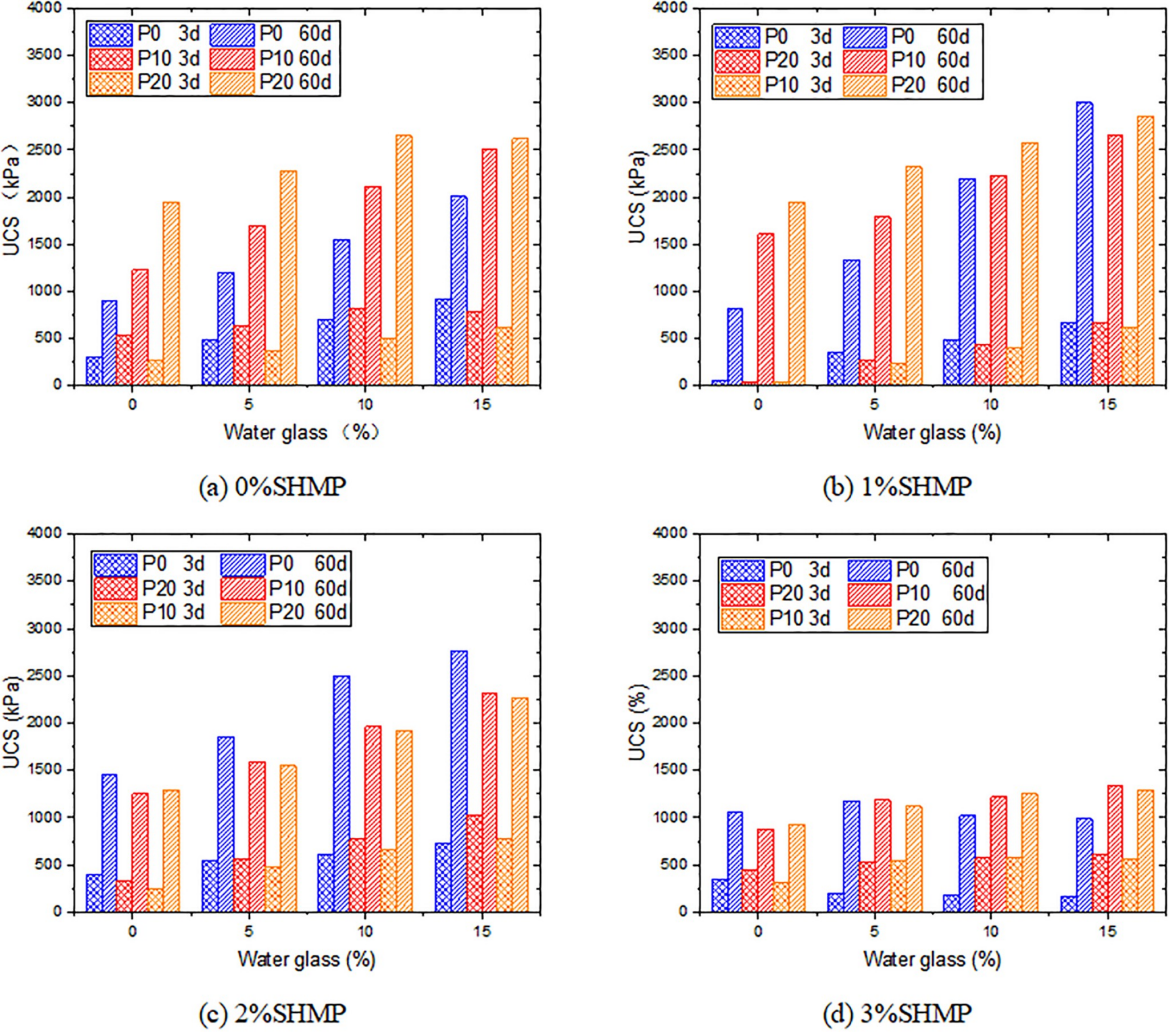
Variation of UCS with SHMP, PG and water glass.

It is well known that adding gypsum into the cement system can generate AFt, thereby delaying the early hydration reaction of cement, but it has a positive effect on improving the strength in the later stage [48, 49]. Fig 10(a) shows the impact of PG addition on the strength of samples at different ratios. The 10% PG increased the strength of samples at 3 days, possibly due to the limited retarding effect of minor PG and the reduction of the system’s water content. As PG increased, the UCS of L0P0G0 decreased from 304.3 kPa to 260.6 kPa at 3 days after adding 20% PG. However, at 60 days, the strength increased by over 100% from 894.4 kPa to 1946.5 kPa, which fully demonstrated the strengthening effect of Aft in forming a spatial network structure with other hydration products. Adding both PG and water glass could further enhance the strength, but the strength growth was not simply cumulative and had some attenuation. As the content of water glass increased, the growth-promoting effect of PG on the later strength decreased. We hypothesize that this may be due to the reaction between water glass and Ca^2+^ released from PG, which further increased the amount of ettringite formed. However, an excess of ettringite can cause expansion, which may adversely affect strength development.

In the SHMP system, the strengthening effect of PG on the later stage strength of samples is gradually reduced by the increasing hydration products of cement. At high SHMP content (2% and 3%), PG even had an adverse effect. This is because the addition of PG increased the calcium element content, which resulted in a significant amount of gel formed by the complexation between SHMP and Ca^2+^ in the early stage of samples. It significantly affected the samples’ mechanical property. In addition, an interesting phenomenon was observed at 3% SHMP. Especially in the early stage, the 3-day strengths of L3P0G0~L3P0G15 were 339.1 kPa, 204.1 kPa, 183.4 kPa, and 161.7 kPa, respectively, without the addition of PG, and the strength decreased with the water glass. However, the incorporation of PG resulted in a notable improvement in the strength of samples subsequent to the addition of water glass. For instance, the 3-day strength of L3P10G15 was 610.2 kPa, which was 35% higher than that of L3P10G0 (451.6 kPa). Under high SHMP conditions, the Ca^2+^ from cement chelated in the system, while the addition of PG introduced Ca^2+^ and reacted with sodium silicate to form C-S-H, thereby enhancing the strength. However, this contribution to strength was limited. There was no significant increase in strength in any group after adding PG and water glass at 60 days.

### 3.3 Hydraulic conductivity tests

**Fig 11:**
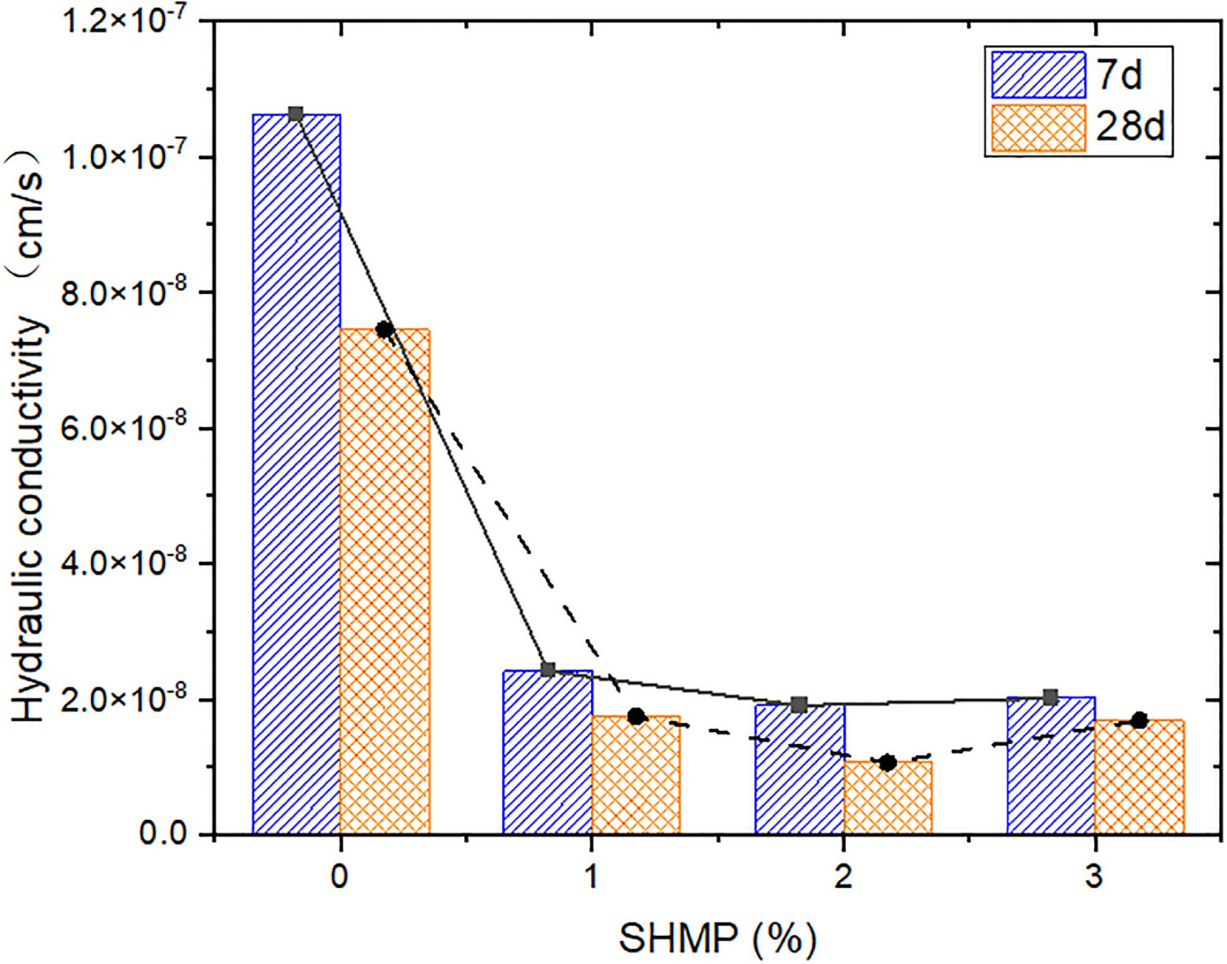
Variation of hydraulic conductivity with SHMP content.

Hydraulic conductivity is a characteristic of the permeability of soil by water and other liquids. When the value is too high, it may cause the movement of soil particles or soil skeleton, resulting in the seepage deformation of the foundation [8]. As shown in Fig 11, there was a significant decrease in hydraulic conductivity with SHMP content. The 7-day hydraulic conductivity of L0P0G0 was 1.06×10^−7^ cm/s, while after the addition of 1%, 2%, and 3% SHMP, the hydraulic conductivities became 2.42×10^−8^ cm/s, 1.91×10^−8^ cm/s, and 2.02×10^−8^ cm/s, respectively, all decreased by one order of magnitude. As a common dispersant, SHMP can make the originally disordered soil particles parallel, further improving the anti-permeability performance of the soil [27, 29]. On the other hand, the gel formed by the complexation of SHMP with Ca^2+^ can also significantly reduce the hydraulic conductivity of the material. However, the anti-permeability performance of the modified soil was also affected by the gel generated by cement hydration. SHMP had an obvious inhibitory effect on cement hydration, so the change in the hydraulic conductivity of the solidified soil was not significant with the increase of SHMP.

**Fig 12:**
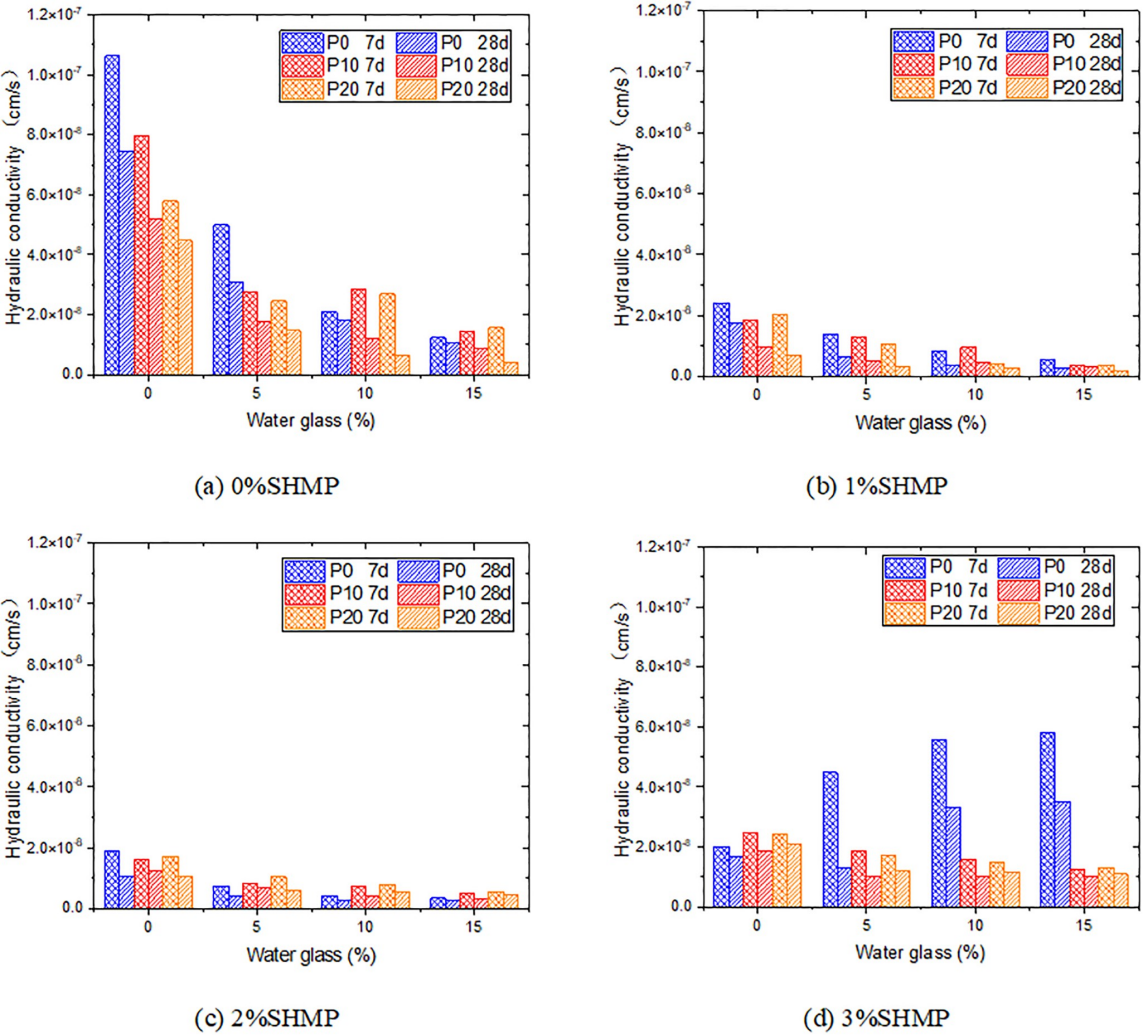
Variation of hydraulic conductivity with additives content.

Fig 12 illustrates the influence of water glass and PG on the hydraulic conductivity at different SHMP content. Similar to UCS tests, when SHMP content was 0%, due to the filling effect of ettringite and the promoting effect of water glass on the hydration of cement, the combination of PG and water glass can make the samples obtain better impermeability in the later stage. But in the groups with 10% and 15% water glass content, the 7d-impermeability of the samples decreased with PG. The hydraulic conductivities of L0P10G15 and L0P20G15 were 1.42×10^−8^ cm/s and 1.57×10^−8^ cm/s, respectively, which were increased by 14.5% and 26.6% compared with L0P0G15 (1.24×10^−8^ cm/s). This may be because the addition of PG increased the concentration of Ca^2+^ in the system, which was unfavorable to the hydration reaction of cement. At the same time, this part of Ca^2+^ may have a competitive reaction with the Ca(OH)_2_, and inhibited the promoting effect of water glass on cement hydration.

The effects of PG and water glass in the system changed constantly with SHMP. At 2% SHMP content, the addition of water glass can still improve the impermeability of the sample, while PG had an adverse effect. For example, the hydraulic conductivity of L2P0G15 was more than 50% higher than that of L2P20G15 at both 7 and 28 days. In the case of 3% SHMP and without PG, the promotion effect of water glass on cement hydration was limited. At the same time, free water was introduced to cause the higher hydraulic conductivity with the water glass content. However, when mixed with PG, the addition of water glass can reduce the hydraulic conductivity of the samples. It can be attributed to the higher concentration of Ca^2+^ after the addition of PG, the reaction with water glass generates calcium silicate hydrate, fills the pores, and improves the anti-permeability of the samples.

**Fig 13:**
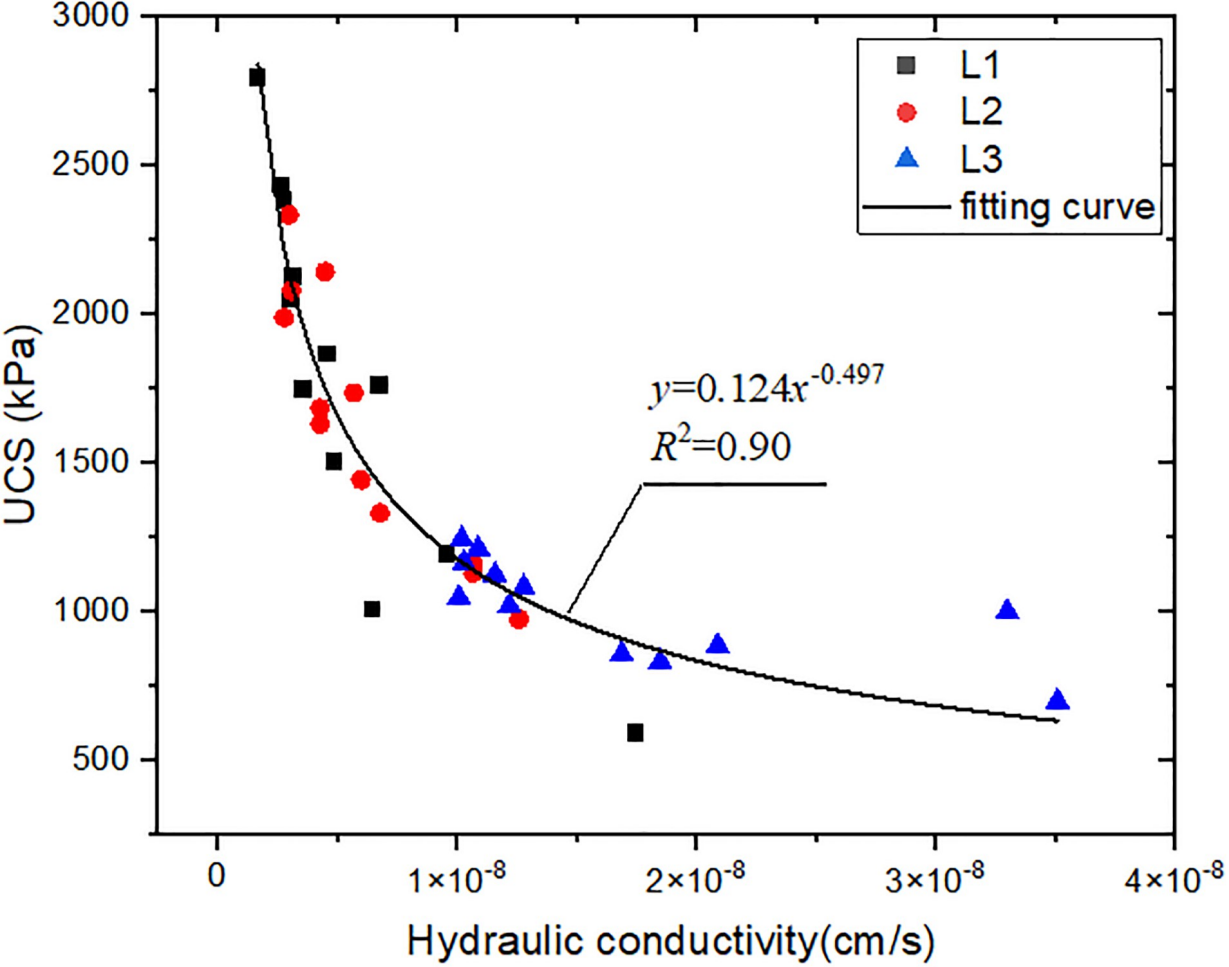
Relationship between UCS and hydraulic conductivity.

Through the regression analysis of L1, L2 and L3 groups by least square fitting method, the relationship between strength and hydraulic conductivity at 28d age can be well fitted by the power function, as shown in Fig 13. When the content of SHMP was low (1%), a significant increase in UCS was observed with a decrease in hydraulic conductivity. However, as the SHMP content increased, the point position of the test data gradually moved to a gentle position, indicating that the relationship between the hydraulic conductivity and strength became unsensitive. SHMP had a bidirectional effect on the material’s impermeability and strength development. It can change the soil particle arrangement, improve impermeability and strength, and promote Ca^2+^ complex formation. However, excessive incorporation can hinder cement hydration, and inhibit the development of strength.

### 3.4 SEM

**Fig 14:**
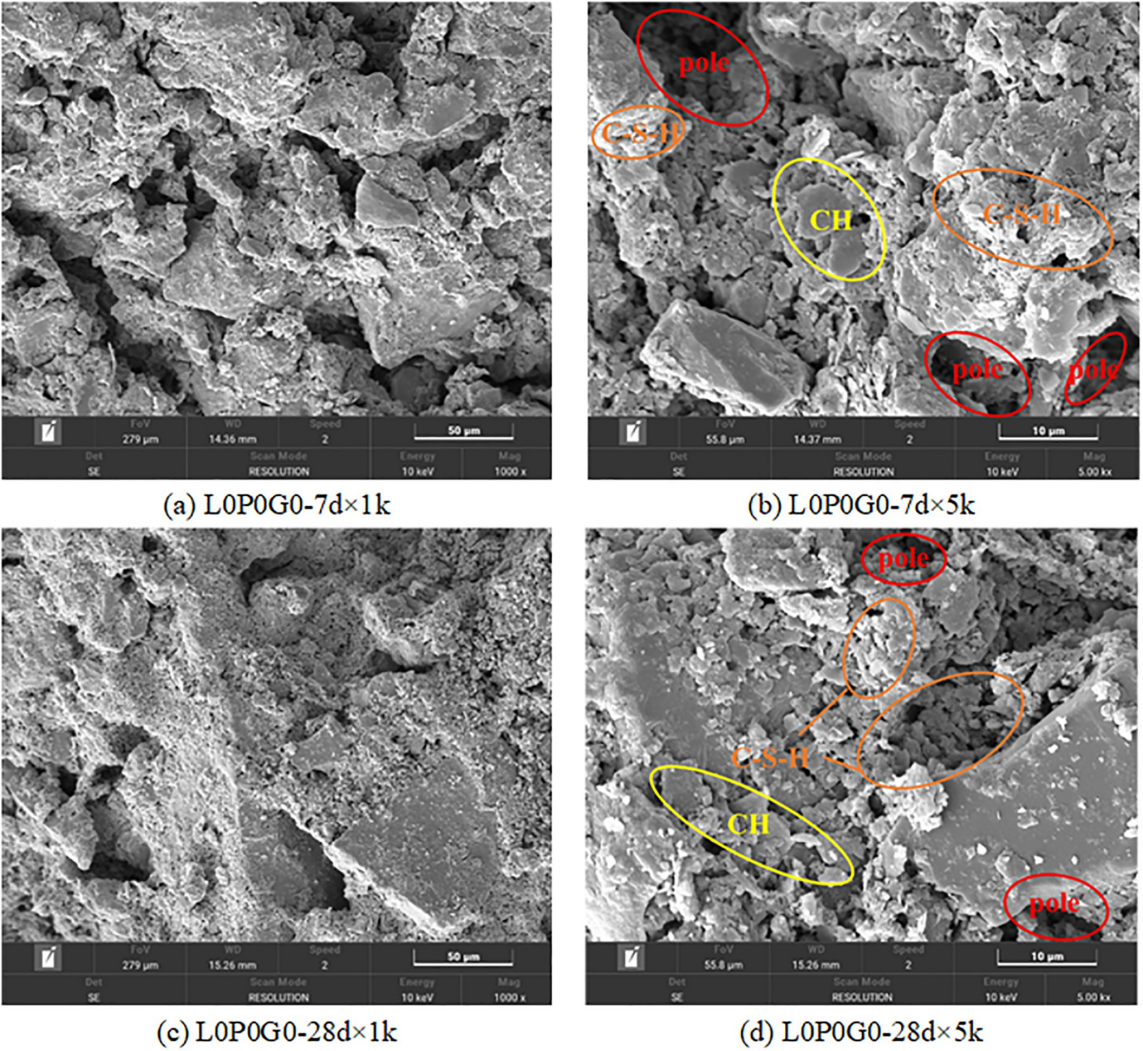
SEM images of different curing time.

Stabilized soil is a cohesive structure formed by bonding soil particles of various shapes with cementitious materials. During the hydration process, crystalline formations and gels were generated, which filled the voids within the soil mass, and improved soil compaction. However, as depicted in Fig 14(a) and 14(b), there were still relatively large and numerous pores between soil particles due to insufficient cement hydration at 7 days. As the hydration reaction of cement continued, the size, shape, and interconnection status of particles were changed. By 28 days, the hydration products almost occupied the inter-particle pores, resulting in a reduction of intergranular pore size and the formation of micro-pores. As a result, the material exhibited a denser structure. It is the main factor contributing to the increase in soil strength and permeability resistance performance.

**Fig 15:**
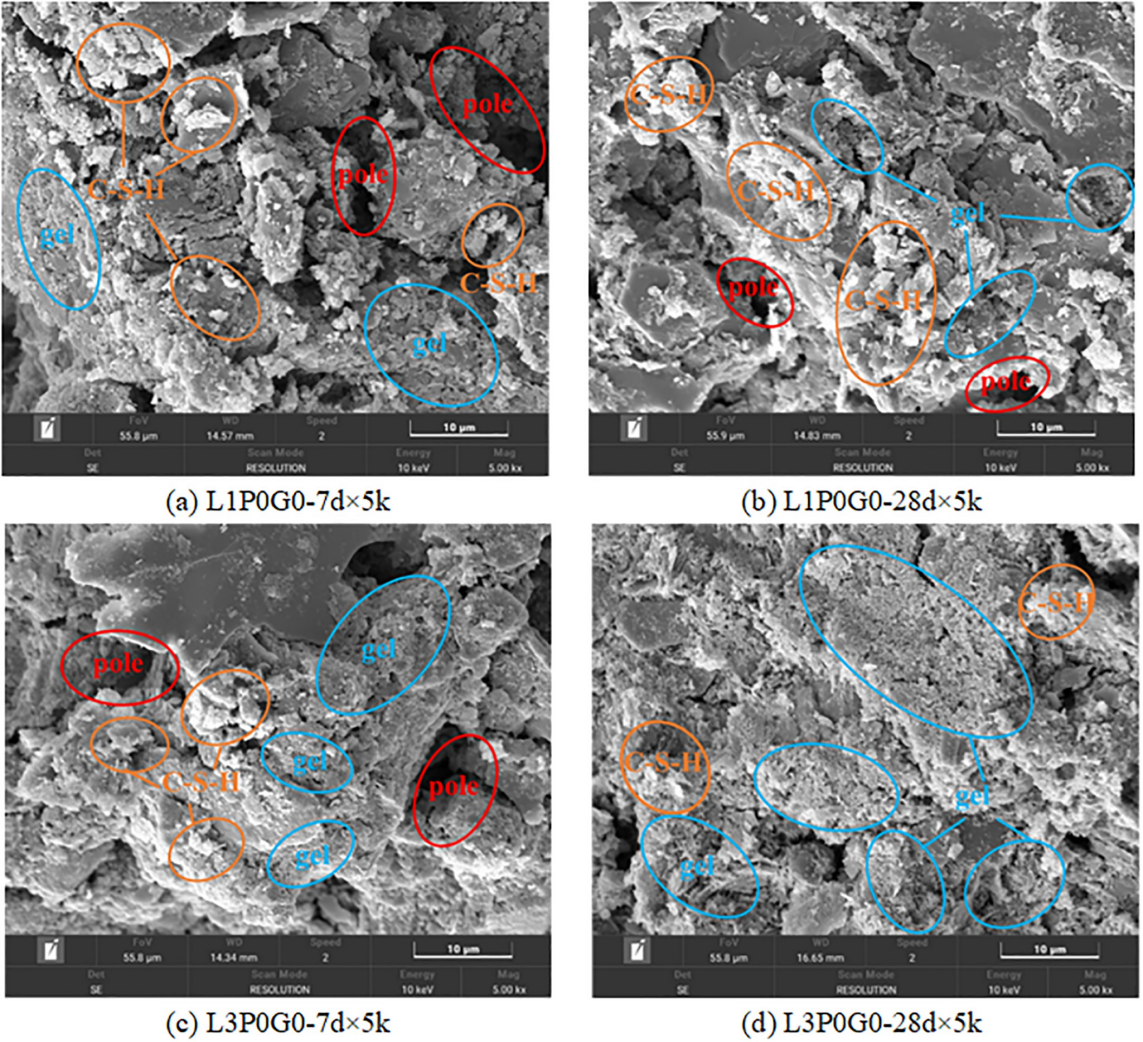
SEM images with different SHMP content.

The addition of SHMP has a significant impact on the hydration process of cement and the development of strength at various ages [50, 51]. The inhibitory effect of SHMP on cement hydration was confirmed by the decrease in cement hydration products at 7-day age with increasing SHMP content, as shown in Fig 15. On other hand, honeycomb-like complexes formed by SHMP and Ca^2+^ could fill the gaps and optimize the particle size distribution between particles to improve the strength and permeability resistance performance. In the L1P0G0 group, the content of this complex is not high enough, resulting in lower strength compared to the L0P0G0 group. At the 28-day age, the negative impact of SHMP on the later stage of cement hydration was evident as the cement hydration products in L1P0G0 were much lower than in L0P0G0 and the significant reduction in strength is manifested on a macro level. In the L3P0G0 group, a large number of complexes formed by SHMP and Ca^2+^ could be observed and they had become the main source of compressive strength. This gel wrapped around soil particles and hydration products, making other hydration products were difficult to observe.

**Fig 16:**
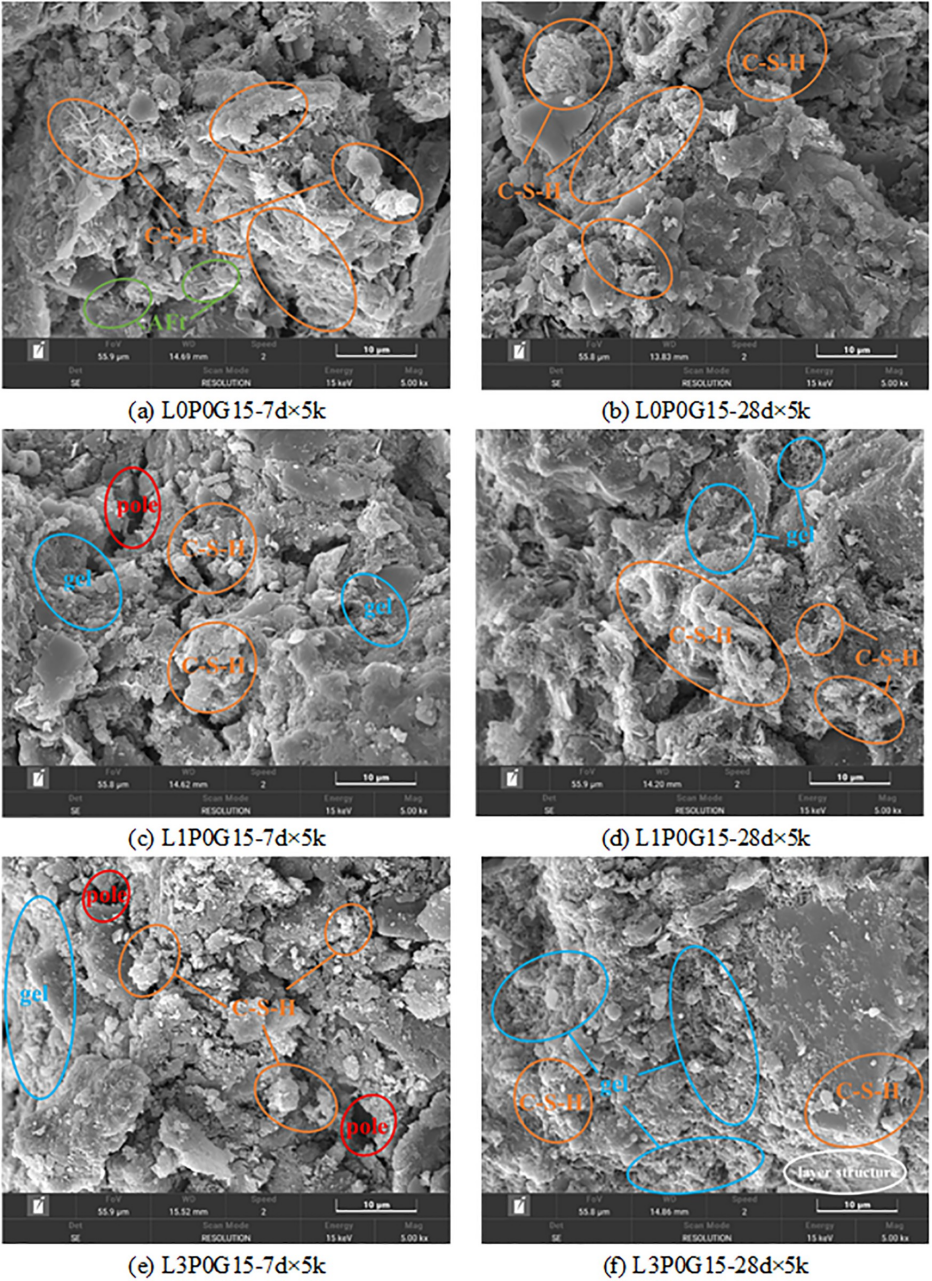
SEM images with different water glass content.

Water glass can significantly promote cement hydration, as evidenced by the rich hydration products observed in the 7-day cured samples (Fig 16(a)). The early-stage UCS of the samples was greatly improved. At 28 days of curing, the cementitious substances in the system continued to grow, leading to a denser internal structure (Fig 16(b)). Comparing Figs 16(c) and 16(d) with 15(a) and 15(b), under 1% SHMP (L1P0G15), the addition of water glass still greatly promoted the hydration process of solidified soil, generating a large amount of hydration products in 7 days. There was a notable increase in the strength at 7 and 28 days compared to L1P0G0. However, when adding 3% SHMP, the promoting effect of water glass was inhibited. As shown in Fig 16(e), the hydration products of cement significantly decreased. Moreover, due to the inhibitory effect of water glass on C_3_A [42], the Ca^2+^ in the system decreased, which greatly reduced the gel formed by the complexation of SHMP and Ca^2+^ in 7 days. After improvement with SHMP, the soil layer exhibited parallel arrangement characteristics in Fig 16(f), with the complexation products serving as connecting fillers. However, the low content of cement hydration products, such as C-S-H, limited the improvement in the unconfined compressive strength (UCS) of the samples.

**Fig 17:**
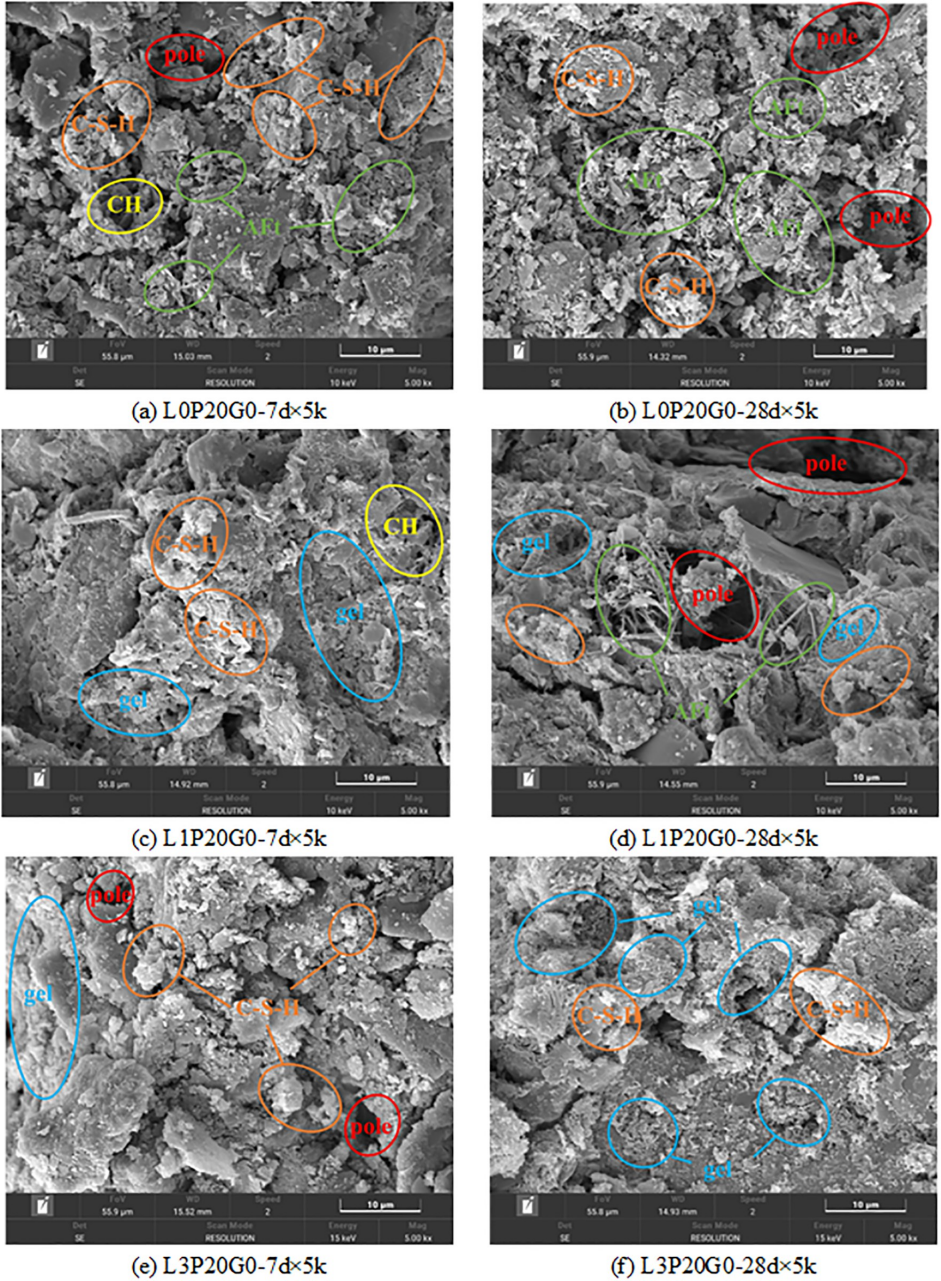
SEM images with different PG content.

As depicted in Fig 17(a), the addition of PG resulted in a high amount of needle-like AFt at 7-day age. These crystals intertwined and penetrated the cement hydration products, forming a spatial network structure that enhanced the samples’ compactness; thus, enhanced the strength and reduced the hydraulic conductivity of the samples. However, due to the retarding effect of PG, there was not much difference in the UCS at 7-day age compared with the L0P0 group. After 28 days, the continuous reaction of cement led to the formation of more abundant hydration products (Fig 17(b)), filling the gaps between particles and continuously improving the mechanical properties of the material. The addition of PG increased the calcium content in the system, leading to the formation of a large amount of gel through the complexation of SHMP and Ca^2+^ at 7-day age. This significantly affected the early performance of the material, as observed in Fig 13(c) and 13(e). However, as the complexation layer continued to form, the cement hydration was inhibited, resulting in the unfavorable effect of PG on the late strength development of L2 and L3 groups. Additionally, AFt with larger length-width ratio were found in the L1P20G0 group (Fig 17(d)), which together with cement hydration products filled the gaps and intersected to form a denser network. This finding explains why the strength of L1P20G0 was about twice that of L1P0G0.

**Fig 18:**
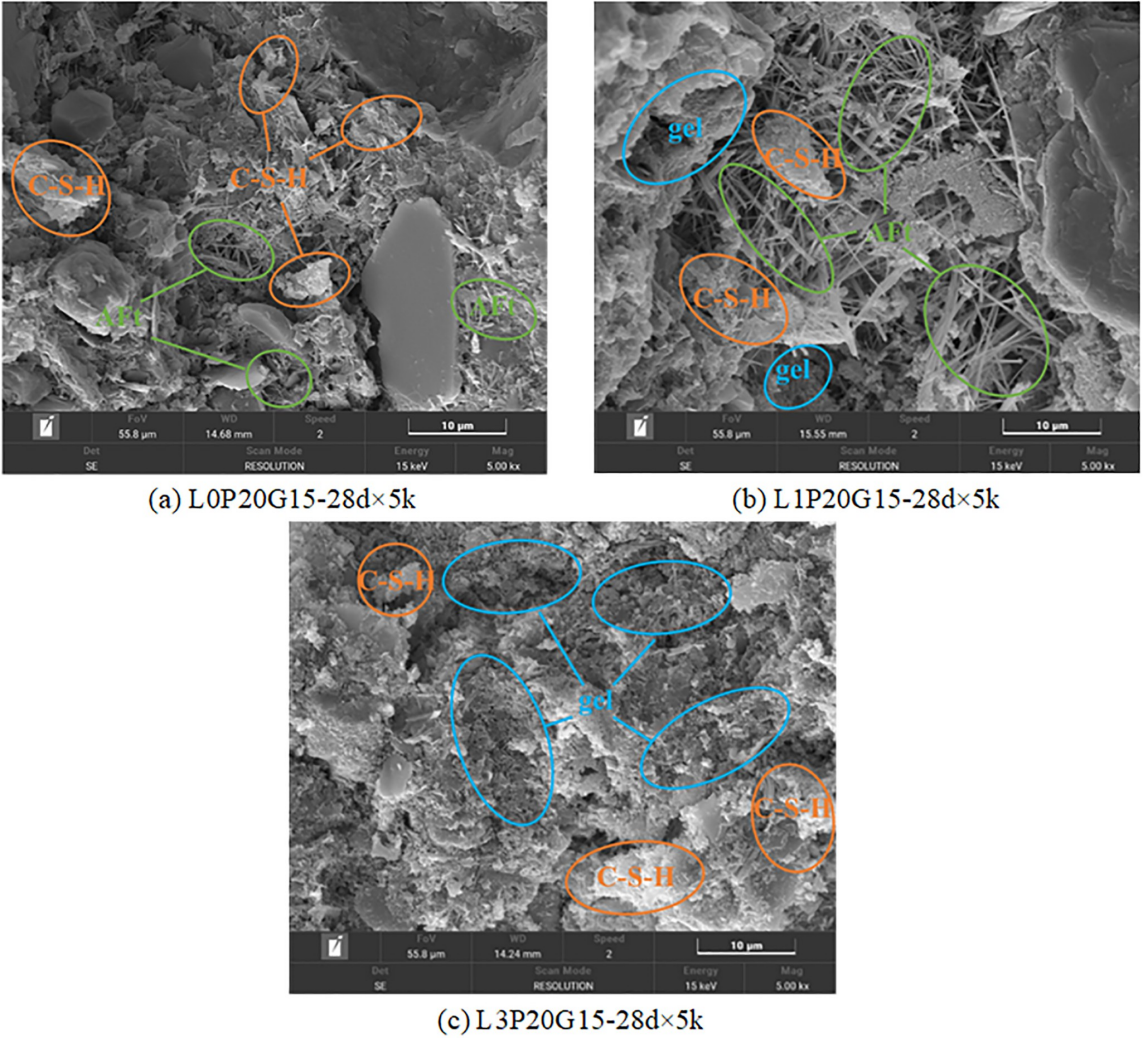
SEM images with different PG and water glass content.

Fig 18 displays the SEM images obtained after adding PG and water glass. It can be observed from Fig 18(a) and 18(b) that a large amount of C-S-H was generated within the sample, and the size of AFt was larger than that observed in Fig 17(b) and 17(d). This indicates that water glass could promote the volume growth of AFt, which was one of the contributing factors for the further increase in strength. However, the absence of AFt in Figs 18(c) and 17(f) implies that its formation was inhibited at higher levels of SHMP [52].

## 4. Conclusion

The novel method for preparing CLSM is proposed with cement-treated construction waste clay. Flow tests, unconfined compressive strength (UCS) tests, and scanning electron microscope (SEM) tests were performed on samples with different SHMP, water glass, and PG. This research effectively addresses the traditional conflict between flowability and strength in clay-based CLSM, providing a balanced solution that enhances both performance metrics. The conclusions can be drawn as follows:

1. The increasing SHMP improved the flowability of the material, but there was an upper limit (2%). The 5% water glass had little effect on the flowability of the CLSM, while the 10% PG improved and maintained the flowability, though excessive amounts of both led to decreased flowability.
2. The SHMP could effectively inhibit the hydration reactions at early stage. However, with the increase in SHMP content, both the early and later strength could be improved by the accumulation of Ca^2+^ and SHMP chelate gel, and the fixation effect on free water in the pores. The maximum compressive strength was obtained at 2% SHMP.
3. With 1% SHMP content, PG and water glass could promote the later strength of the samples. At 2% SHMP content, PG had an adverse effect on the strength of the samples. When the SHMP content increased to 3%, water glass began to diminish the samples’ strength.
4. While SHMP significantly enhanced permeability resistance, its inhibitory effect on cement hydration resulted in minimal change in hydraulic conductivity with increasing dosage, and lower SHMP levels (1%) facilitated local AFt formation, whereas water glass promoted AFt growth in the cement system.

Future research should focus on several key areas to deepen our understanding of CLSM produced from cement-treated construction waste clay. First, investigating the long-term performance and durability of this material under various environmental conditions will offer essential insights into its sustainability. Additionally, establishing quantitative relationships between different soil types and mix ratios will provide valuable information for optimization, enabling customized solutions for a range of applications.

## Supporting information

S1 Data (XLSX)

S1 File (RAR)
